# Identification of Pediatric Retrocecal Appendicitis Using Point of Care Ultrasound (POCUS)

**DOI:** 10.24908/pocusj.v10i01.17744

**Published:** 2025-04-15

**Authors:** Carl Kaplan, Raizada Vaid, Michael Secko

**Affiliations:** 1 Department of Pediatrics, Renaissance School of Medicine at Stony Brook University, Stony Brook, NY, USA; 2 Department of Emergency Medicine, Upstate Medical University, Syracuse, NY, USA; 3 Department of Emergency Medicine, Renaissance School of Medicine at Stony Brook, NY, USA

**Keywords:** Abdominal point of care ultrasound, appendicitis, pediatric abdominal pain, pediatric emergency medicine

## Abstract

Acute appendicitis is the most common pediatric surgical emergency. Diagnosis may be made by targeted point of care ultrasound (POCUS) of the right lower quadrant (RLQ) abdomen. This can be performed by trained emergency physicians and has similar accuracy to ultrasound performed by radiology technologists and interpreted by radiologists (RADUS) [1,2]. Pediatric patients with appendicitis may present without classical clinical signs and symptoms. Retrocecal appendicitis is often diagnosed late at perforation due to the anatomical position limiting diagnosis with ultrasound, despite the high prevalence of retrocecal appendix as an anatomic variation (up to 65%). Given the limited sensitivity for ultrasound in the diagnosis of appendicitis in patients with retrocecal appendix, these patients often undergo advanced imaging with computed tomography (CT) or magnetic resonance imaging (MRI), especially when increased abdominal wall thickness and/or high Body Mass Index (BMI) further limit the ultrasound examination [4–6]. We present a case series of retrocecal appendicitis imaged and diagnosed with POCUS, using novel transducer and patient positioning. In addition to standard graded compression of the RLQ with POCUS, this technique may add to the diagnostic accuracy of patients presenting atypically with anatomic variants.

## Novel Technique

**Figure 1.**
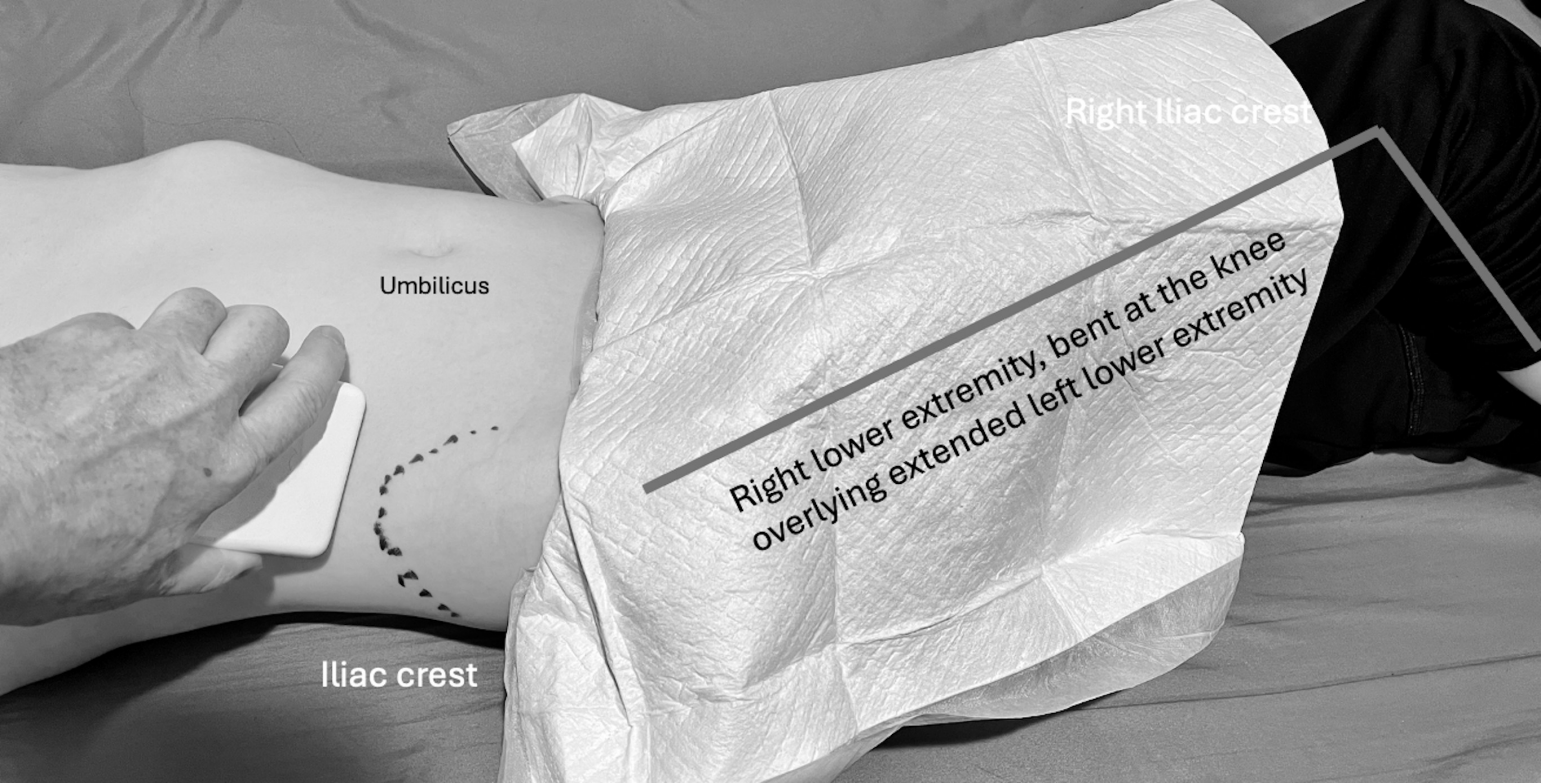
Patient in partial left lateral decubitus with novel technique.

This case series was collected in a dedicated pediatric emergency department (ED) of a children's hospital within a general hospital. Ultrasound performed by radiology technologists and interpreted by radiologists (RADUS) was available and interpreted around the clock by pediatric radiologists or those trained in body imaging. Point of care ultrasound (POCUS) may be used at the discretion of the provider to hasten the diagnostic evaluation and employ the appropriate treatments and resources. All caregivers consented and patients assented to POCUS and positioning, as necessary for the examination. Each patient underwent right lower quadrant (RLQ) POCUS using graded compression of the abdominal wall with a Philips CX50 (Andover, MA, USA) L12-3 linear transducer. When no appendix was visualized with the patient supine, patients were asked to cross their right leg over the left knee, tilting the right hemipelvis anteriorly and creating a partial left lateral decubitus position. The transducer was then positioned superior to the right iliac crest in a transverse plane and angled inferiorly into the pelvis (novel technique). When pathology was visualized, orthogonal views and transducer repositioning were employed to acquire adequate images. (Figure 1)

## Case 1

**Figure 2.**
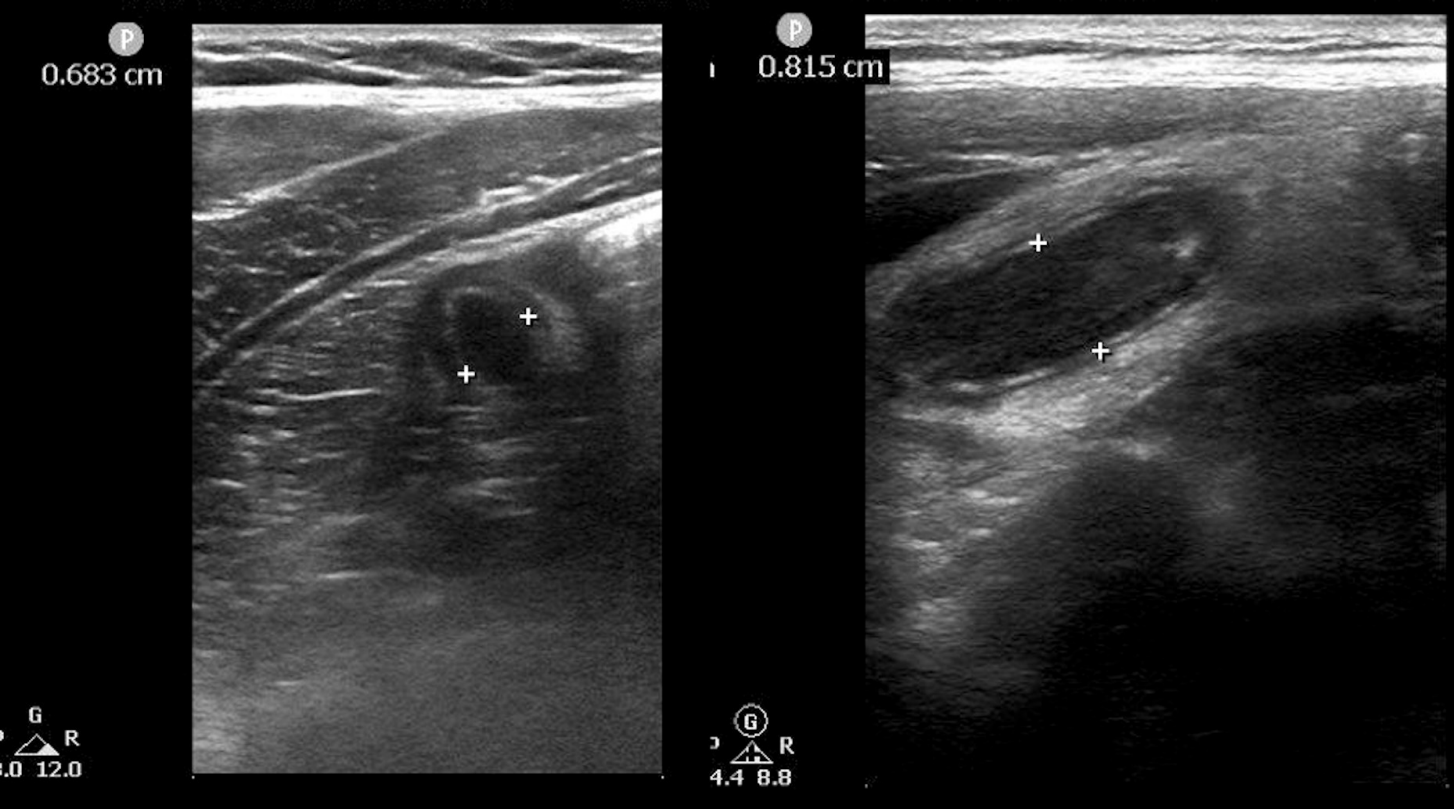
Left – Case 1. Short axis image of dilated appendix with wall thickening. adjacent cecum. Right – Case 2. Long axis image of dilated appendix with increased echogenicity of surrounding soft tissue.

A 16-year-old girl without significant past medical history presents to the ED with RLQ abdominal pain for one day, radiating to the umbilicus. The pain was worse with right leg movement and walking – associated with one episode of non-bloody, non-bilious emesis. The patient's vital signs included temperature 37.1 °C, pulse 104, respiratory rate 18, and blood pressure 113/78, and she appeared well. The remainder of the patient exam was only remarkable for localized RLQ tenderness superior to McBurney's point and midline suprapubic tenderness to palpation, without rebound or guarding. Laboratory results included serum WBC 12.9K (ANC 11.5K), beta HCG negative, urinalysis unremarkable, and Pediatric Appendicitis Risk Calculator (pARC) score 50%. POCUS using graded compression did not identify appendicitis with a standard approach. POCUS with repositioning revealed a retrocecal fluid-filled, non-compressible blind-ending appendix measuring 6.8 mm in diameter with wall thickening. (Figure 2)

RADUS was unable to visualize appendix. Computed tomography (CT) abdomen/pelvis with intravenous contrast, suggested by consultants, was interpreted as acute appendicitis. The diagnosis was confirmed by surgical pathology following uncomplicated laparoscopic appendectomy. The patient was discharged on postoperative day #1.

## Case 2

A 12-year-old boy without significant past medical history presents to the ED with a two-day history of post-prandial RLQ pain, associated with episodic non-bloody, non-bilious emesis. The patient was afebrile, and vital signs included temperature 37.1 °C, pulse 83, respiratory rate 15, and blood pressure 115/68. He appeared well and the exam was only remarkable for localized tenderness to deep palpation in the RLQ abdomen, immediately lateral to McBurney's point, without rebound and guarding. Laboratory studies included WBC 10.7, ANC 9, urinalysis unremarkable, and pARC score 29%. POCUS employing the novel technique revealed a retrocecal, non-compressible appendix, measuring 8 mm in diameter, with increased echogenicity of the peri-appendiceal soft tissue (Figure 2). RADUS identified a fluid-filled, non-compressible 8 mm diameter appendix with wall thickening and a small amount of free fluid surrounding. The patient was admitted to the pediatric surgery service. During laparoscopic appendectomy, the patient was noted to have perforated appendicitis. This patient had an uncomplicated hospital course, and was discharged on postoperative day # 2. The diagnosis was confirmed with surgical pathology.

## Case 3

**Figure 3.**
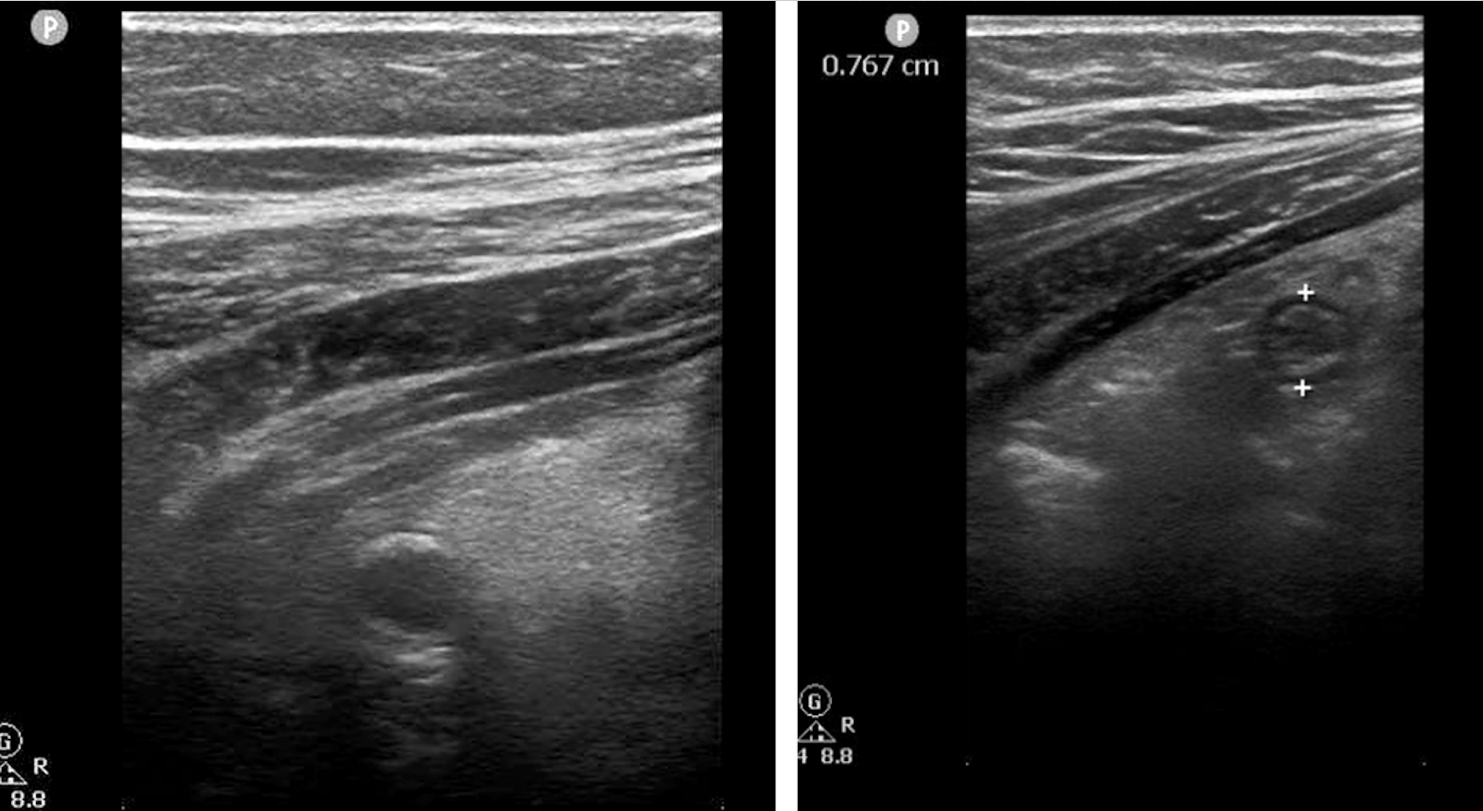
Case 3. Short axis images, at different levels showing dilated appendix with thickened walls and increased echogenicity of surrounding soft tissue.

A 9-year-old boy without significant past medical history presents to the ED with 3 hours of acute RLQ pain associated with subjective fever. The patient reported improvement following pre-hospital acetaminophen administration, and he had no additional symptoms. Upon arrival to the ED the patient had a temperature of 36.4o C, pulse 110, respiratory rate 18, and blood pressure 128/82. He appeared well and the physical exam was only remarkable for tenderness to deep palpation of the RLQ without guarding or rebound. Laboratory studies included serum WBC 16.6 (ANC 11.5), normal urinalysis, and pARC score 42%. POCUS using the novel technique showed retrocecal appendicitis (Figure 3).

RADUS identified a dilated, noncompressible, fluid-filled tubular structure, 1.3 cm in length with multiple shadowing calcific structures. CT scan was suggested by consultants and showed dilation of a retrocecal appendix, 1.2 cm with calcified appendicolith in the midportion of the appendix and surrounding inflammatory changes. The findings were confirmed with surgical pathology. The patient had an uncomplicated course and was discharged on postoperative day # 0.

## Summary

Appendicitis in the pediatric population may present atypically with anatomic variations leading to delays in diagnosis and potential exposure to medical radiation through testing. While classical RADUS was able to identify appendicitis in two of three cases reported, the novel technique may have utility when strong clinical suspicion of appendicitis exists and classical RLQ ultrasound technique is non-diagnostic. The novel POCUS technique may offer a sonologist enhanced capability to identify retrocecal appendices early and without additional resources – as it did in all cases described. Prospective studies with the novel technique are needed to assess the accuracy and feasibility of this modality in the pediatric ED.
